# Early hypocortisolism with persistent remission following osilodrostat in a patient with long-standing Cushing disease

**DOI:** 10.1210/jcemcr/luag033

**Published:** 2026-03-18

**Authors:** Liat Sasson, Ilan Shimon

**Affiliations:** Institute of Endocrinology, Rabin Medical Center, Beilinson Hospital, Petach Tikva 49100, Israel; Gray Faculty of Medical and Health Sciences, Tel Aviv University, Tel Aviv 69978, Israel; Institute of Endocrinology, Rabin Medical Center, Beilinson Hospital, Petach Tikva 49100, Israel; Gray Faculty of Medical and Health Sciences, Tel Aviv University, Tel Aviv 69978, Israel

**Keywords:** adrenal insufficiency, cortisol, Cushing disease, osilodrostat

## Abstract

Cushing syndrome is a disorder of endogenous hypercortisolism characterized by increased morbidity and mortality; when surgery is not curative or feasible, medical therapies targeting pituitary adrenocorticotropic hormone or adrenal cortisol production are essential. We report a case of early-onset hypocortisolism and sustained remission following a brief osilodrostat therapy in a 70-year-old woman with Cushing disease who had been treated for many years with pasireotide and metyrapone. Ten days after initiating osilodrostat, she developed clinical signs of adrenal insufficiency and a low morning serum cortisol of 2.8 µg/dL (SI: 76 nmol/L) (reference range 7-25 µg/dL [SI: 193-690 nmol/L]); osilodrostat was discontinued, and glucocorticoid replacement was initiated, remaining glucocorticoid-replacement dependent at low doses for 2 months. Over subsequent follow-up of over 20 months, her 24-hour urinary free cortisol normalized, and she maintained persistent biochemical and clinical eucortisolism off all Cushing therapy, with no relapse of hypercortisolism. She also experienced weight loss of 16.5 kg and marked improvement in diabetes control, enabling discontinuation of insulin and glucagon-like peptide-1 (GLP-1) receptor agonist therapy. This is among the earliest documented cases of osilodrostat-induced hypocortisolism with long sustained hormonal remission after treatment discontinuation, emphasizing the need for early monitoring and prolonged follow-up.

## Introduction

Cushing disease is a rare disorder of chronic hypercortisolism caused by an adrenocorticotropic hormone (ACTH)-secreting pituitary adenoma, associated with increased morbidity and mortality [1]. Transsphenoidal surgery is the first-line treatment, but recurrence is common, and medical therapy plays a crucial role [2]. Pasireotide, a multireceptor somatostatin analog, is the only pituitary-directed drug addressing the underlying ACTH hypersecretion, approved by the US Food and Drug Administration (FDA) [3]. Osilodrostat is a potent, orally administered 11β-hydroxylase inhibitor, FDA approved for adults with Cushing syndrome for whom surgery is not an option or not curative [2, 3]. Here we describe an unusual case of rapid-onset hypocortisolism after brief osilodrostat therapy, resulting in sustained biochemical remission.

## Case presentation

A 70-year-old woman was diagnosed with ACTH–dependent Cushing disease in 2008, with typical clinical features and biochemical confirmation of hypercortisolism. Magnetic resonance imaging at diagnosis demonstrated a cystic pituitary macroadenoma measuring approximately 15 mm in maximal diameter. The patient declined transsphenoidal surgery and was managed medically.

She received long-term treatment with pasireotide (1200 mcg twice daily), achieving partial biochemical control, and subsequently required adjunctive metyrapone due to persistent hypercortisolism. During prolonged pasireotide therapy, serial pituitary imaging demonstrated marked tumor shrinkage, with maximal diameter reduced to less than 4 mm, as previously reported [4].

## Diagnostic assessment

Pasireotide and metyrapone combination therapy was continued for several years with fluctuating 24-hour urinary free cortisol (UFC) levels. Prior to initiation of osilodrostat, biochemical evaluation demonstrated persistent hypercortisolism. The last morning serum cortisol measured before treatment initiation was 44.3 µg/dL (SI: 1223 nmol/L) (reference range, 7-25 µg/dL [SI: 193-690 nmol/L]). UFC was elevated at 129 µg/24 hours (SI: 356.6 nmol/24 hours) (upper limit of normal, 63 µg/24 hours [SI: 173 nmol/24 hours]).

Computed tomography of the adrenal glands performed during follow-up demonstrated no structural abnormalities, with no evidence of adrenal enlargement or nodularity.

## Treatment

On February 2024, osilodrostat 2 mg twice daily was initiated due to persistent hypercortisolism, and metyrapone was discontinued at that time. Within 10 days, she developed fatigue, nausea, and hypotension; morning cortisol was low, 2.8 µg/dL (SI: 76 nmol/L), consistent with adrenal insufficiency. Osilodrostat was stopped, and prednisone 10 mg daily was started with rapid clinical improvement. She continued prednisone replacement therapy at 4 to 5 mg daily for 2 months, followed by gradual tapering down for an additional month. No adrenal crises occurred and adrenal imaging was normal.

## Outcome and follow-up

**Figure 1:**
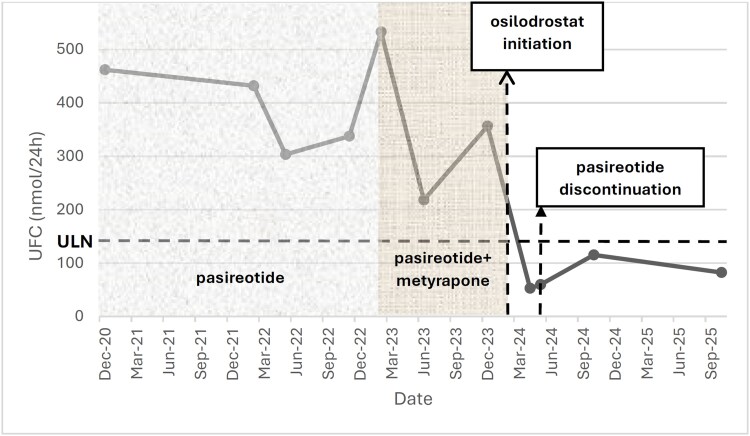
Twenty-four-hour urinary free cortisol levels over time and treatment phase. Longitudinal 24-hour urinary free cortisol (UFC) measurements demonstrating biochemical changes during sequential medical therapies for Cushing disease. Shaded regions indicate treatment intervals with pasireotide monotherapy, combination pasireotide–metyrapone therapy, and the brief 10-day course of osilodrostat, followed by the post-osilodrostat period. A horizontal dashed line represents the upper limit of normal for UFC in SI units (173 nmol/24 hours; conventional units, 62.7 μg/24 hours). A rapid decline in UFC following osilodrostat initiation and sustained eucortisolism thereafter are shown.

Approximately 5 weeks after osilodrostat discontinuation, UFC had normalized (19 µg/24 hours [SI: 52 nmol/24 hours]) and remained within the normal range on repeated measurements over more than 20 months of follow-up (Fig. 1). During the first 3 months of follow-up, the patient was receiving physiologic prednisone replacement therapy; however, prednisone was withheld for 24 to 48 hours prior to each UFC and morning serum cortisol assessment.

Two months after osilodrostat initiation, pasireotide was also discontinued.

**Figure 2:**
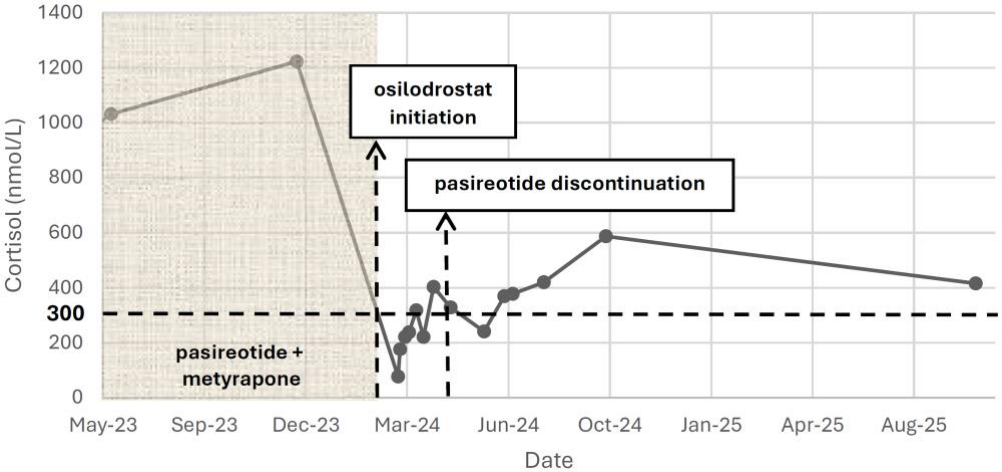
Changes in serum cortisol levels before and after osilodrostat therapy. Longitudinal morning serum cortisol levels from May 2023 to August 2025. The shaded region denotes the period of pasireotide–metyrapone therapy prior to osilodrostat initiation. A sharp decline in serum cortisol is observed immediately after the start of osilodrostat therapy (first vertical dashed line), leading to profound hypocortisolism within days. The second vertical dashed line indicates subsequent discontinuation of pasireotide. The dashed horizontal line represents the lower limit of the normal morning serum cortisol reference range in SI units (300 nmol/L; conventional units, 10.9 μg/dL) for comparison. Following brief osilodrostat exposure, cortisol levels gradually increased but remained subnormal for several months, with gradual biochemical recovery during long-term follow-up.

**Figure 3:**
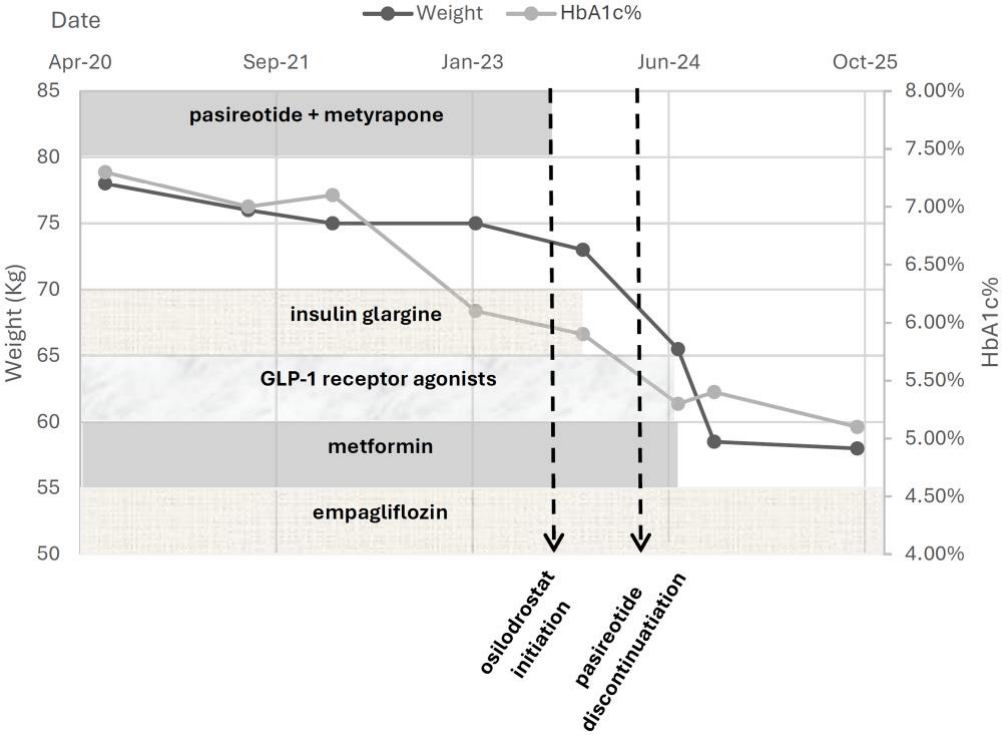
Changes in body weight and hemoglobin A1c over time in relation to Cushing and diabetes therapies. A dual-axis plot illustrating longitudinal changes in body weight (left axis) and hemoglobin A1c (HbA1c; right axis) from 2020 to 2025. Background shaded bands depict diabetes treatments over time (insulin glargine, glucagon-like peptide-1 [GLP-1] receptor agonists, metformin, and empagliflozin), while the upper band marks the period of pasireotide–metyrapone therapy for Cushing disease. Two vertical dashed lines indicate key clinical events: initiation of osilodrostat (February 2024) and subsequent discontinuation of pasireotide. A marked reduction in weight and HbA1c occurred following the short 10-day exposure to osilodrostat, enabling discontinuation of insulin, metformin, and GLP-1 receptor agonist therapy, with maintenance on oral therapy alone (empagliflozin). The figure highlights significant and sustained metabolic improvement during follow-up despite cessation of Cushing-directed treatment.

Morning cortisol levels showed a gradual but incomplete recovery, reaching subnormal values about 1 month after osilodrostat discontinuation and remaining stable thereafter (Fig. 2). Plasma ACTH measured during follow-up in August 2024 was 78 pg/mL (SI: 17.2 pmol/L) (reference range, 7-63 pg/mL [SI: 1.6-13.9 pmol/L]). At the last follow-up (October 2025) morning cortisol was 15.1 µg/dL (SI: 416 nmol/L) with UFC of 29.8 µg/24 hours (SI: 82.2 nmol/24 hours). Following the brief osilodrostat exposure, while off any Cushing therapy, the patient experienced a marked metabolic improvement with a 16.5-kg weight loss (from 75 to 58.5 kg) and significant improvement in glycemic control. Insulin, metformin and glucagon-like peptide-1 (GLP-1) receptor agonist therapy were discontinued, and her diabetes remained well controlled only by empaglifozin 10 mg daily, with a hemoglobin A1C (HBA1C) level of 5.1% (reference range, <5.7%) at last follow-up (Fig. 3).

## Discussion

Transsphenoidal surgery remains the treatment of choice for Cushing disease; however, up to 30% to 40% of patients have persistent or recurrent hypercortisolism following surgery and require medical therapy [1]. Adrenal steroidogenesis inhibitors, including metyrapone, ketoconazole, levoketoconazole, mitotane, and the more recently approved osilodrostat, suppress cortisol synthesis at different enzymatic steps [5]. Osilodrostat is a potent, orally active inhibitor of 11β-hydroxylase (CYP11B1) and, to a lesser extent, aldosterone synthase (CYP11B2), leading to inhibition of the final step of cortisol and aldosterone biosynthesis [2, 6]. Compared with older adrenal-blocking drugs, osilodrostat has a rapid onset of action, predictable pharmacokinetics, and sustained cortisol suppression, which make it highly effective for medical management of Cushing disease.

The pivotal phase 3 LINC 3 study showed normalization of UFC in 66% of patients at 34 weeks, along with improvements in blood pressure, glucose metabolism and weight loss [2]. These benefits persisted in the long-term extension, confirming durable efficacy. Subsequent trials, including LINC 4, and real-world reports have confirmed osilodrostat's safety and effectiveness, though adrenal insufficiency is reported in 15% to 40% of patients [7-9]. These events are typically reversible upon dose reduction or drug withdrawal [6].

Osilodrostat-induced adrenal insufficiency results from potent blockade of CYP11B1, leading to rapid cortisol reduction and compensatory ACTH stimulation with increased 11-deoxycortisol levels [6]. Most patients recover normal adrenal function within days or weeks of dose adjustment. However, several recent reports have described prolonged adrenal insufficiency after discontinuation of osilodrostat, lasting several months, suggesting either sustained enzymatic inhibition or secondary adrenal atrophy [7-9]. Ferrière et al [7] reported 2 patients with severe and prolonged adrenal insufficiency persisting up to 9 months after drug cessation, hypothesizing continued inhibitory effects on adrenal steroidogenesis. Similarly, Poirier et al [8] described prolonged adrenocortical blockade after short-term osilodrostat exposure, with adrenal recovery only after several months. Castinetti et al [9] emphasized that delayed or prolonged hypocortisolism should be closely monitored, particularly during dose titration and after discontinuation.

Our case is unique in demonstrating both *very early-onset* adrenal insufficiency, occurring within 10 days of treatment initiation, and *exceptionally prolonged* eucortisolism lasting more than 20 months after discontinuation. Moreover, pasireotide treatment was discontinued to improve cortisol secretion. Such rapid onset is rare at the initial dose of 2 mg twice daily, suggesting increased sensitivity or cumulative adrenal suppression. Several possible mechanisms may explain this unique response. One contributing factor is prolonged ACTH suppression from many years of pasireotide therapy, which may have reduced the adrenal glands responsiveness at baseline. Although historical serial ACTH measurements are not available, a plasma ACTH level obtained during follow-up after osilodrostat discontinuation was markedly elevated (78 pg/mL [SI: 17.2 pmol/L]) in the presence of low-normal serum cortisol concentrations. This biochemical pattern resembles primary adrenal insufficiency and may reflect impaired adrenal steroidogenic capacity following long-standing ACTH suppression. In addition, the preceding metyrapone exposure likely provided an added degree of 11β-hydroxylase blockade before osilodrostat was initiated. A further possibility is an intrinsic susceptibility of adrenal CYP11B1 to persistent inhibition [10]. Together, these factors may have produced a profound and durable impairment of cortisol synthesis.

An additional notable finding was the patient's substantial clinical improvement, including a 16.5-kg weight reduction and resolution of diabetes requiring discontinuation of insulin, metformin and GLP-1 therapy. These benefits parallel those reported in pervious trials where normalization of cortisol levels correlated with improvements in metabolic parameters and quality of life [2, 10, 11]. The persistence of biochemical remission raises the intriguing hypothesis that osilodrostat may, in rare instances, induce long-term functional remission of hypercortisolism through sustained suppression of residual tumor or adrenal responsiveness.

From a practical perspective, this case highlights several important considerations for clinicians. Adrenal insufficiency may occur abruptly, even at low starting doses of osilodrostat, emphasizing the need for careful initiation and early follow-up. Close biochemical monitoring during the first weeks of treatment is essential to promptly identify excessive cortisol suppression. In clinical practice, the decision to continue osilodrostat with glucocorticoid replacement or to interrupt therapy should be individualized, based on the severity and persistence of clinical and biochemical hypocortisolism. Mild cases may be managed with glucocorticoid supplementation, whereas more severe or prolonged manifestations may warrant treatment discontinuation. Moreover, recovery of adrenal function after discontinuation can be markedly delayed, warranting prolonged follow-up and patient education regarding symptoms of adrenal insufficiency. Finally, in some patients, sustained cortisol suppression may not only reflect overtreatment or drug persistence but could also indicate a more durable remission of Cushing disease activity [10].

In summary, osilodrostat represents a major advance in the medical management of Cushing disease, providing effective and sustained control of hypercortisolism. However, clinicians should remain vigilant for both early and prolonged adrenal insufficiency, particularly in patients with prior exposure to pituitary or adrenal directed therapies. Careful dose titration, patient education, and long-term biochemical surveillance are crucial to ensure safety and identify cases that may progress to sustained remission.

## Learning points

- Osilodrostat can induce adrenal insufficiency as early as 10 days after initiation.
- Prolonged cortisol suppression may persist beyond 1 year despite drug discontinuation.
- Prior pituitary and adrenal suppression may increase vulnerability.
- Long-term biochemical and clinical monitoring is essential.
- Sustained hypocortisolism may overlap with remission and may require replacement therapy.

## Contributors

Both authors made individual contributions to authorship. L.S. and I.S. were involved in the conception and design of the work and in the acquisition, analysis, and interpretation of the clinical data. L.S. drafted the manuscript, and I.S. critically reviewed it for important intellectual content. Both authors approved the final version of the manuscript and agree to be accountable for all aspects of the work.

## Data Availability

Original data generated and analyzed for this case report are included in this published article.
